# Perinatal Characteristics and Automated Auditory Brainstem Response Screening Outcomes in Newborns: A Retrospective Hospital-Based Cohort Study

**DOI:** 10.3390/healthcare14213631

**Published:** 2026-10-26

**Authors:** Ali Bestami Kepekçi, Ahmet Hamdi Kepekçi

**Affiliations:** 1 Department of Anesthesia, Vocational Health High School, Istanbul Yeni Yüzyıl University, Istanbul 34010, Turkey; 2 Department of Otorhinolaryngology, Faculty of Medicine, Istanbul Yeni Yüzyıl University, Istanbul 34010, Turkey; dr.kepekci@gmail.com

**Keywords:** automated auditory brainstem response, neonatal intensive care unit, newborn hearing screening, gestational age, loss to follow-up, perinatal risk factors, programmatic audit, referral

## Abstract

**Background:** Universal newborn hearing screening (UNHS) programs are essential for early detection of congenital hearing impairment. In community and secondary hospital settings, distinguishing true screening failures from protocol-driven risk referrals and tracking outpatient follow-up represent key operational challenges. **Objective:** To evaluate automated Auditory Brainstem Response (AABR) screening outcomes and explore their associations with delivery mode, anesthesia type, nationality, and selected perinatal characteristics in a retrospective newborn cohort. Secondary objectives were to describe protocol-driven risk referrals despite normal screening results and to assess incomplete screening due to loss to outpatient follow-up. **Methods:** This retrospective observational cohort study evaluated 406 consecutive singleton newborns undergoing universal AABR screening between 1 June 2024 and 31 May 2025 at a private secondary hospital in Istanbul, Türkiye. Screenings followed a multi-stage protocol using the MADSEN AccuScreen system (35 dB nHL click stimuli). Outcomes were classified into four mutually exclusive groups: (1) normal AABR without referral, (2) protocol-driven referral due to predefined hearing-loss risk factors despite a normal AABR, (3) final AABR refer (screening failure), and (4) incomplete screening/lost to outpatient follow-up. Primary exploratory analyses compared completed normal passes (*n* = 384) with final screen refers (*n* = 7) using exact tests. A post hoc sensitivity analysis was conducted treating all incomplete cases as screening refers (*n* = 22 vs. *n* = 384). **Results:** Among 406 singleton newborns, 343 (84.5%) passed AABR without referral, 41 (10.1%) passed AABR but were referred for audiological surveillance due to clinical risk factors, 7 (1.7%) had a final AABR refer result and were referred for diagnostic audiological evaluation, and 15 (3.7%) had an initial refer but defaulted before completing outpatient rescreening. In the primary analysis, lower gestational age showed a nominal association with a final AABR refer [median 37.0 weeks (IQR 36.5–38.0) vs. 38.0 weeks (IQR 38.0–39.0); *p* = 0.026]. In the sensitivity analysis incorporating all 15 incomplete cases (*n* = 22), this nominal association with lower gestational age was also observed [median 37.5 weeks vs. 38.0 weeks; *p* = 0.028]. Neonatal intensive care unit (NICU) admission (*p* = 0.018) and predefined risk factors (*p* = 0.004) showed nominal univariate associations with screening refer; after Holm adjustment for multiple comparisons, only the predefined risk factor composite remained below 0.05. Sex, delivery mode (cesarean vs. vaginal), and anesthesia type were not significantly associated with screening outcome; however, given the small number of events (*n* = 7), these findings should be regarded as statistically inconclusive rather than definitive evidence of no effect. Among the 41 risk-referred infants, prolonged NICU hospitalization (>5 days) was the predominant referral criterion (*n* = 29, 70.7%). In the post hoc worst-case sensitivity analysis, general anesthesia compared with regional anesthesia (*p* = 0.014) and Syrian nationality compared with Turkish nationality (*p* = 0.008) showed nominal associations with the hypothetical expanded refer outcome, illustrating the sensitivity of these findings to incomplete follow-up. **Conclusions:** In this retrospective hospital-based cohort, only seven newborns had a final AABR refer result, limiting the precision of association estimates. Although lower gestational age and NICU admission showed nominal associations with final screening refer, only the predefined hearing-loss risk factor composite remained statistically significant after adjustment for multiple comparisons. Fifteen newborns (3.7%) did not complete outpatient rescreening, identifying a measurable gap in follow-up documentation. These findings support improved screening-completion tracking and warrant validation in larger cohorts with confirmatory audiological outcomes.

## 1. Introduction

Neonatal hearing loss is one of the most common sensory disorders in childhood and, if not diagnosed and treated early, can lead to lasting negative effects on language development, communication skills, and cognitive processes [1]. Newborn hearing screening tests are critical for the early diagnosis and intervention of permanent hearing loss. Since hearing forms the basis of language development and cognitive abilities, universal screening programs are implemented early and are standardized in many countries.

Permanent childhood hearing loss detected through universal newborn hearing screening has an estimated pooled prevalence of approximately 1.1 per 1000 screened newborns. A systematic review and meta-analysis reported a higher prevalence among NICU infants (5.9 per 1000) than among infants not admitted to the NICU (0.8 per 1000) [2]. These findings emphasize the importance of appropriate surveillance and follow-up for high-risk newborns. Congenital hearing loss following neonatal hearing screening has also been investigated in previous cohort studies [3].

Universal newborn hearing screening programs, as part of Early Hearing Detection and Intervention (EHDI) systems, play a critical role in the early diagnosis and management of hearing loss in infants. Otoacoustic Emission (OAE) and Auditory Brainstem Response (ABR) tests, commonly used in screening programs, provide objective assessment of auditory function in newborns. These methods form the core components of EHDI programs [4,5]. Automated Auditory Brainstem Response (AABR), in particular, enables objective assessment of auditory pathway responses and is widely used in newborn hearing screening programs, while diagnostic ABR is used as part of the confirmatory audiological evaluation of suspected hearing loss [6,7].

The main perinatal and neonatal risk factors associated with adverse hearing outcomes include prematurity, low birth weight, low Apgar scores, prolonged NICU hospitalization, mechanical ventilation, hyperbilirubinemia, sepsis, and craniofacial anomalies [8,9]. Importantly, newborns with recognized hearing-loss risk factors may require audiological follow-up even when the initial hearing screening result is normal. Therefore, referral based on predefined clinical risk factors should be distinguished from referral following an abnormal or suspicious screening result. This distinction is particularly important when evaluating screening outcomes and referral patterns within universal newborn hearing screening programs.

In recent years, the potential effects of delivery mode (cesarean or vaginal) and anesthesia techniques (spinal, general, etc.) on neonatal physiological stability and auditory screening outcomes have also been investigated. Because anesthetic agents can cross the placenta and may exert transient effects on the neonatal central nervous system, they have been proposed as potential determinants of newborn hearing screening outcomes [10,11,12]. Güler et al. reported that delivery mode and anesthesia type may affect newborn ABR screening results; specifically, significant associations were observed between the duration of fetal exposure to anesthetic agents, the time from uterine incision to delivery, and initial ABR screening results among infants delivered under general anesthesia [13]. However, evaluating these associations requires consideration of the number of screening failures and the availability of detailed anesthesia exposure data.

Beyond biological and perinatal determinants, sociodemographic characteristics and migration-related factors may also contribute to variability in newborn hearing screening outcomes. Differences in prenatal care access, socioeconomic conditions, and perinatal risk profiles among migrant populations have been proposed as possible explanations for these variations. Çıkrıkçı et al. reported higher AABR referral rates among Syrian refugee newborns than among newborns from Türkiye [14].

In this context, the primary aim of the present study was to evaluate AABR screening outcomes and explore their associations with delivery mode, anesthesia type, nationality, and selected perinatal characteristics in a retrospective hospital cohort. Given the limited number of final screening failures, these analyses were considered exploratory rather than confirmatory. A secondary aim was to describe referral patterns for further audiological assessment, distinguishing referral following a final AABR screening failure from protocol-driven referral based on predefined hearing-loss risk factors despite a normal AABR result. The study also evaluated newborns with incomplete screening due to loss to outpatient follow-up, providing a descriptive assessment of the hospital screening and referral process.

## 2. Materials and Methods

### 2.1. Study Design and Sampling

This retrospective observational cohort study was conducted at a private hospital in Istanbul, Türkiye. The reporting of this retrospective observational cohort study was guided by the Strengthening the Reporting of Observational Studies in Epidemiology (STROBE) guidelines [15]. The study population consisted of newborns born between 1 June 2024 and 31 May 2025 who underwent universal newborn hearing screening. Demographic and clinical data were retrospectively obtained from electronic medical records.

A total of 410 records were initially identified during the study period. During data quality assessment, four duplicate records, representing administrative re-registrations of the same newborns, were identified by cross-checking medical record numbers and maternal identifiers and subsequently removed, leaving 406 unique newborns in the final analytic dataset. Review of the obstetric records confirmed that all 406 newborns were singleton births, with no multiple births identified. No missing values were identified in the variables included in the reported analyses; therefore, no newborns were excluded because of missing data.

**Figure 1:**
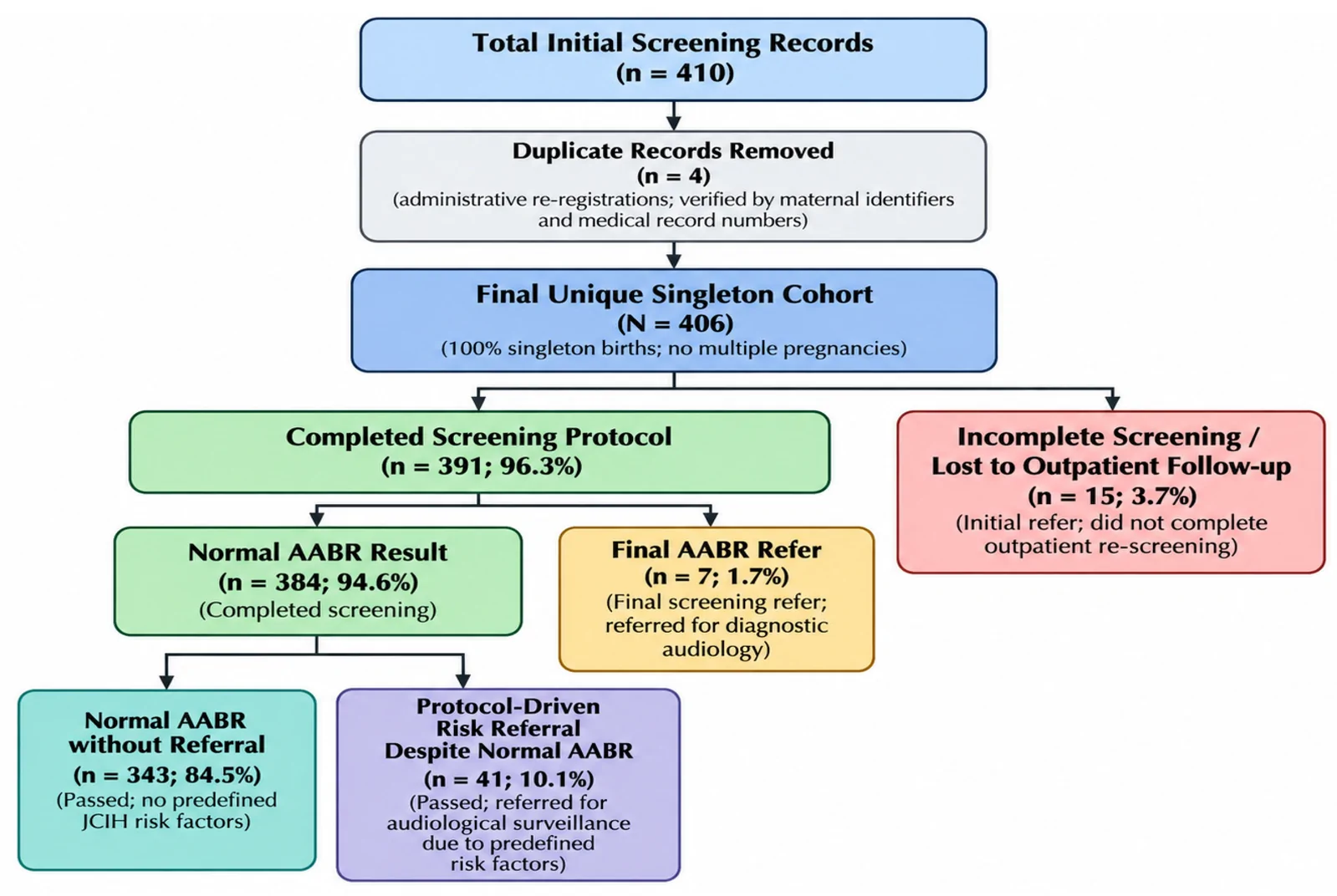
Flow diagram of cohort selection and newborn AABR screening outcomes. Note: All percentages are calculated using the total cohort (N = 406). AABR: automated auditory brainstem response; JCIH: Joint Committee on Infant Hearing.

Of the 406 newborns, 391 (96.3%) completed the screening protocol, while 15 (3.7%) did not complete outpatient rescreening. Among those completing screening, 384 had a normal AABR result, and 7 had a final AABR refer. Of the 384 newborns with normal AABR results, 343 required no further referral, whereas 41 were referred for audiological follow-up because of predefined clinical risk factors. The study population and screening outcomes are summarized in Figure 1.

The study cohort was defined by the availability of an AABR screening record rather than by the total number of live births at the hospital. Consequently, the dataset did not permit a reliable determination of the number of newborns who were not screened, were transferred before screening, or underwent screening at another institution. Screening coverage among all hospital births could therefore not be assessed.

All eligible newborns with accessible clinical records were included, and no additional sample size calculation was performed because the study retrospectively included all eligible newborns with available AABR screening records during the predefined study period.

### 2.2. Inclusion and Exclusion Criteria

All live-born infants who underwent AABR hearing screening within the first month of life were eligible for inclusion. Infants with major congenital ear anomalies that could interfere with reliable hearing screening and those with incomplete or inaccessible medical records were considered ineligible. No newborns were excluded because of major congenital ear anomalies or missing data in the variables included in the reported analyses. All 406 newborns included in the study were singleton births.

### 2.3. Data Collection

Demographic and clinical data were retrospectively obtained from the electronic medical record system. The variables collected included the following:

- Maternal age
- Mode of delivery (vaginal or cesarean)
- Anesthesia method (regional, general, or no anesthesia)
- Sex
- Birth weight (grams)
- Gestational age (weeks)
- Apgar scores (1st and 5th minute)
- NICU admission status and duration
- History of ototoxic drug use and mechanical ventilation
- Family history of hearing loss
- Nationality (Türkiye, Syria, other)

Risk factors for hearing loss were evaluated as prolonged NICU hospitalization (>5 days), family history of hearing loss, ototoxic drug use, need for mechanical ventilation, maternal infection history, craniofacial anomalies (e.g., preauricular pit), and syndromic disorders. These risk factors were evaluated separately from the AABR screening result because their presence could result in referral for further audiological evaluation despite a normal AABR result. The predefined risk-factor composite was based on the risk indicators documented in the hospital database and did not constitute a comprehensive assessment of all risk indicators included in the 2019 Joint Committee on Infant Hearing (JCIH) recommendations [16].

NICU admission status was recorded as a separate clinical variable, whereas NICU hospitalization lasting more than 5 days was considered a predefined risk criterion for audiological follow-up.

### 2.4. AABR Test

Newborn hearing assessment was performed using automated auditory brainstem response (AABR) screening with the MADSEN AccuScreen device (Otometrics, Taastrup, Denmark). All screenings were conducted in a quiet room while the newborn was naturally asleep, and no sedation was administered. The device’s standard automated screening protocol was used, employing click stimuli at approximately 35 dB nHL. Screening results were recorded as either “pass” or “refer”.

The unit of analysis was the individual newborn; bilateral “Pass” was considered a normal screening result, whereas “Refer” in either ear was classified as a screening refer.

Screening was performed according to the institutional multi-stage newborn hearing screening protocol. Newborns who did not pass the initial screening (T1) underwent repeat screening (T2), either before discharge or during a scheduled outpatient visit. Infants who did not pass the second screening were scheduled for a final screening assessment (T3). Those who continued to receive a “Refer” result at the final screening stage were referred for diagnostic audiological evaluation.

In accordance with the institutional newborn hearing screening protocol, newborns with predefined hearing-loss risk factors could be referred for further audiological evaluation despite a normal AABR result. Therefore, the AABR screening result and the subsequent referral decision were treated as distinct outcomes in the present study.

For study purposes, screening and referral outcomes were classified into four mutually exclusive categories: (1) normal AABR without referral, (2) referral because of predefined hearing-loss risk factors despite a normal AABR result, (3) final AABR refer following completion of the screening protocol, and (4) incomplete screening/lost to outpatient follow-up. The latter category included 15 newborns who received a “Refer” result during initial screening but did not return for scheduled outpatient rescreening, leaving their final screening status unknown. The distribution of these groups is presented in Figure 1.

For analyses of the AABR screening result itself, newborns with incomplete screening were excluded from the primary analysis. Newborns with a normal AABR result who were subsequently referred solely because of predefined hearing-loss risk factors were retained in the normal AABR group because their screening result itself was not abnormal. A post hoc sensitivity analysis was also performed by classifying all 15 incomplete cases as screening refers.

Infants requiring confirmatory diagnostic audiological evaluation were referred to external tertiary audiology centers. As post-referral diagnostic records were not available in the hospital’s electronic medical record system, confirmatory audiological outcomes could not be evaluated in this study.

### 2.5. Ethical Approval

This retrospective observational study was conducted in accordance with the ethical principles of the Declaration of Helsinki. The study used routinely collected clinical records from the hospital’s newborn hearing screening program. No additional intervention or procedure was performed for research purposes. Ethical approval for the retrospective use of these clinical records was obtained from the local ethics committee on 5 July 2025 (Decision No. 2025/49). Written informed consent for routine newborn hearing screening and clinical care was obtained from the newborns’ legal guardians before screening. No additional research-specific informed consent was obtained from the newborns’ legal guardians. The retrospective use of existing clinical records was approved by the local ethics committee.

### 2.6. Statistical Analysis

Data were analyzed using IBM SPSS Statistics version 26.0 (IBM Corp., Armonk, NY, USA) and Python scientific computing libraries (SciPy 1.17.0). Continuous variables are presented as mean ± standard deviation (SD) or median and interquartile range (IQR), as appropriate. Categorical variables are expressed as frequencies and percentages.

Baseline characteristics were summarized across the four screening and referral outcome groups. For the primary analysis, newborns who completed the screening protocol (*n* = 391) were evaluated. The 384 newborns with a normal AABR result, including 41 referred because of predefined hearing-loss risk factors, were compared with the 7 newborns with a final AABR refer result. Categorical variables were compared using Fisher’s exact test for 2 × 2 tables and the Fisher–Freeman–Halton test for larger contingency tables, as appropriate. These analyses were conducted using the statistical software specified above, with particular attention to the small number of outcome events. Conditional maximum-likelihood odds ratios with exact 95% confidence intervals were calculated for sparse 2 × 2 comparisons where estimable. Continuous and ordinal variables were compared using the two-sided Mann–Whitney U test, with asymptotic *p*-values adjusted for tied ranks. Given the small number of final AABR refer events and the presence of tied observations, these results were interpreted as exploratory.

An exploratory minimum detectable effect (MDE) analysis was conducted to characterize the sensitivity of the primary comparisons. Using two-sided Fisher’s exact tests, a significance level of 0.05, 80% statistical power, the observed group sizes, and fixed reference-group event proportions, the minimum detectable absolute difference was approximately 6.7 percentage points for vaginal versus cesarean delivery and 13.7 percentage points for general versus regional anesthesia. These calculations were conditional on the assumed reference event rates and were intended to illustrate the study’s limited ability to detect smaller differences, rather than to establish confirmatory statistical power.

Given the small number of final AABR refer events (*n* = 7), the primary analysis was considered exploratory. No multivariable regression was performed for the primary screening outcome because of the risk of overfitting and unstable parameter estimates. To address multiple testing, the Holm–Bonferroni procedure was applied to the 12 primary comparisons. Both nominal and multiplicity-adjusted findings were interpreted cautiously, with emphasis on absolute event frequencies, effect estimates, and confidence intervals.

A post hoc sensitivity analysis was performed to assess the potential impact of incomplete outpatient follow-up. Under a worst-case assumption, all 15 newborns with incomplete screening were classified as screening refers, resulting in 22 assumed refer outcomes and 384 normal AABR results among the total cohort of 406 newborns. Comparisons were repeated using Fisher’s exact test and the Mann–Whitney U test, as appropriate. This analysis represented a hypothetical scenario rather than observed final screening outcomes.

A two-sided *p*-value < 0.05 was considered nominally statistically significant.

## 3. Results

### 3.1. Study Population

The final analytic dataset comprised 406 unique newborns, including 205 females (50.5%) and 201 males (49.5%). Citizens of Türkiye accounted for 251 newborns (61.8%), Syrian nationals for 57 (14.0%), and other nationalities for 98 (24.1%). Delivery was by cesarean section in 309 newborns (76.1%) and vaginally in 97 (23.9%).

Mean maternal age was 29.6 ± 5.7 years and mean birth weight was 3268.7 ± 441.0 g. Median gestational age was 38 weeks (IQR 38–39). Median 1-min and 5-min Apgar scores were 10 (IQR 8–10) and 10 (IQR 9–10), respectively. Thirty-six newborns (8.9%) were admitted to the NICU, and 46 (11.3%) had at least one predefined hearing-loss risk factor.

Of the 406 newborns, 391 (96.3%) completed the screening protocol. Among these, 343 (84.5%) had a normal AABR result without referral, 41 (10.1%) had a normal AABR result but were referred because of predefined hearing-loss risk factors, and 7 (1.7%) had a final AABR refer result. The remaining 15 newborns (3.7%) did not complete outpatient rescreening, leaving their final screening status unknown. The distribution of screening outcomes is presented in Figure 1.

### 3.2. AABR Screening and Referral Outcomes

Among the 46 newborns with at least one predefined hearing-loss risk factor, 41 had a normal AABR result and were referred under the risk-based follow-up protocol, 4 had a final AABR refer result, and 1 did not complete outpatient rescreening. The 15 incomplete screening cases were retained as a separate descriptive category and excluded from the primary AABR comparison.

The baseline maternal, obstetric, and neonatal characteristics of the four outcome groups and the total cohort are presented in Table 1.

### 3.3. Factors Associated with the AABR Screening Result

After exclusion of the 15 newborns with incomplete outpatient follow-up, 391 newborns were included in the analysis of the AABR screening result: 384 had a normal result, and 7 had a final AABR refer result (Table 2).

Male sex was not associated with a final AABR result (OR 2.55, 95% CI 0.41–27.05; *p* = 0.284). Comparisons involving delivery mode (*p* = 0.673) and anesthesia type (overall exact *p* = 0.791) were statistically inconclusive because of the small number of final AABR refer events.

The distribution of AABR results differed nominally across nationality groups (exact *p* = 0.047). However, this finding was based on only seven final AABR refer outcomes, and no refer cases occurred in the heterogeneous “Other” nationality category. The corresponding category-specific estimates were therefore imprecise and should be interpreted cautiously.

A final AABR refer result occurred in 3 of 35 newborns admitted to the NICU (8.6%), compared with 4 of 356 newborns without NICU admission (1.1%), corresponding to an unadjusted OR of 8.16 (95% CI 1.15–50.55; *p* = 0.018). The presence of at least one predefined hearing-loss risk factor showed a similar nominal association (OR 11.02, 95% CI 1.80–77.88; *p* = 0.004). Both estimates had wide confidence intervals, reflecting the limited number of final AABR refer outcomes. These findings also represent overlapping clinical signals because prolonged NICU hospitalization contributed to the predefined risk factor composite.

Neither the 1-min nor the 5-min Apgar score differed between newborns with normal and final AABR refer results (*p* = 0.887 and *p* = 0.706, respectively). Median gestational age was lower among newborns with a final AABR refer result [37 weeks (IQR 36.5–38)] than among those with a normal result [38 weeks (IQR 38–39); *p* = 0.026]. Birth weight was lower in the final AABR refer group, but the between-group difference did not reach nominal statistical significance (*p* = 0.138) (Table 2).

After Holm–Bonferroni correction for 12 primary comparisons, only the predefined hearing-loss risk factor composite remained statistically significant (adjusted *p* ≈ 0.048). All other nominal associations did not survive multiplicity adjustment.

### 3.4. Distribution of Risk Factors Among Protocol-Driven Referrals

Among the 406 newborns, 41 (10.1%) had a normal bilateral AABR screening result but were referred for further audiological follow-up because of predefined hearing-loss risk factors. The distribution of referral criteria is presented in Table 3.

Prolonged neonatal intensive care unit (NICU) hospitalization (>5 days) was the most frequently recorded referral criterion (*n* = 29, 70.7%), followed by a family history of permanent childhood hearing loss (*n* = 7, 17.1%) and craniofacial anomalies (preauricular pit/tag; *n* = 3, 7.3%). Ototoxic medication exposure, prolonged mechanical ventilation, maternal infection or disease, and syndromic disorders were each recorded in one infant (2.4%).

Some newborns had more than one risk factor; therefore, the percentages do not sum to 100%. These referrals were based on predefined clinical risk criteria rather than an abnormal AABR screening result. No inferential comparisons were performed within this subgroup because the risk factors themselves determined referral status.

### 3.5. Post Hoc Sensitivity Analysis: Impact of Incomplete Screening Cases

Fifteen newborns (3.7% of the total cohort) did not complete the recommended outpatient rescreening pathway and were therefore excluded from the primary analysis of final AABR screening outcomes.

To assess the potential impact of these incomplete cases, a post hoc worst-case sensitivity analysis was performed in which all 15 newborns were hypothetically classified as having a final AABR refer result. Under this assumption, the number of refer outcomes increased from 7 to 22, corresponding to 5.4% of the total cohort (22/406), compared with 1.7% (7/406) observed in the original cohort.

In this hypothetical analysis, lower gestational age (*p* = 0.028), general anesthesia compared with regional anesthesia (*p* = 0.014), and Syrian nationality compared with Turkish nationality (*p* = 0.008) showed nominal associations with the expanded refer outcome. NICU admission (*p* = 0.120) and the presence of at least one predefined hearing-loss risk factor (*p* = 0.090) were not statistically significant (Table 4).

These findings demonstrate that the associations observed in the primary analysis are sensitive to assumptions regarding incomplete follow-up. The hypothetical classification does not represent confirmed screening failure or diagnosed hearing loss, and the sensitivity analysis should therefore be interpreted as exploratory rather than confirmatory.

## 4. Discussion

UNHS programs aim to facilitate early identification of congenital hearing impairment while ensuring appropriate diagnostic referral and follow-up. In this one-year retrospective audit of 406 singleton newborns undergoing AABR screening, 7 infants (1.7%) had a final AABR refer result, 41 (10.1%) passed screening but required risk-factor-based audiological follow-up, and 15 (3.7%) did not complete outpatient rescreening. In the primary exploratory analysis, lower gestational age, NICU admission, and predefined hearing-loss risk factors showed nominal associations with final AABR refer results. However, after Holm–Bonferroni correction for multiple comparisons, only the predefined risk factor composite remained statistically significant. Delivery mode, anesthesia type, infant sex, and Apgar scores were not significantly associated with the primary screening outcome.

Lower gestational age showed a nominal association with final AABR refer results. Infants with a final refer had a median gestational age of 37 weeks, compared with 38 weeks among those with normal screening results (*p* = 0.026). Previous studies have identified gestational age as a potentially relevant factor in newborn hearing-screening outcomes [17,18,19,20]. In a large cohort of 76,460 newborns, Yang et al. reported a nonlinear association between gestational age and initial hearing-screening referral, with the lowest referral odds occurring at approximately 39 weeks [18]. These findings suggest that gestational maturity may influence early screening performance. Nevertheless, the initial screening outcomes evaluated by Yang et al. are not directly equivalent to the final AABR outcomes assessed in our study. Transient conductive factors and developmental differences in auditory pathway maturation may contribute to variation in screening responses, although these mechanisms were not directly investigated in our cohort [6,7]. Given the small number of final refer events and the absence of statistical significance after multiplicity adjustment, the observed association with gestational age should be considered exploratory rather than evidence of an independent predictive effect.

NICU admission was nominally associated with a higher final AABR refer proportion (8.6% versus 1.1%; *p* = 0.018). Similarly, newborns with at least one predefined hearing-loss risk factor had a higher refer proportion than those without such factors (8.9% versus 0.9%; *p* = 0.004). Previous investigations have reported less favorable hearing-screening outcomes among newborns requiring intensive care [14,19,20]. Li et al. reported higher AABR referral rates among NICU infants than among well-baby nursery infants [19], while other studies have highlighted the contribution of neonatal clinical vulnerability to screening outcomes [20]. In our cohort, NICU admission and the predefined risk factor composite were not independent clinical exposures: prolonged NICU hospitalization was a major component of the risk-factor definition. Among the 41 newborns referred despite normal AABR results, 29 (70.7%) met the criterion of NICU hospitalization lasting more than five days (Table 3). Consequently, these associations should be interpreted as overlapping clinical signals rather than separate causal effects. The JCIH 2019 recommendations emphasize AABR-based screening and continued audiological surveillance for infants with relevant NICU-related risk factors [16].

Neither delivery mode nor anesthesia type showed a statistically significant association with final AABR screening outcome. A previous study conducted at the same center using transient evoked otoacoustic emissions (TEOAEs) similarly reported no significant association with delivery mode or maternal anesthesia technique [12]. Importantly, the present 2024–2025 cohort is completely independent and does not overlap with our earlier 2018–2019 study population [12]; our hospital transitioned from TEOAE to AABR screening protocol to enhance screening accuracy and reduce false-positive referral rates. In contrast, Guler et al. reported associations between anesthesia-related exposure characteristics and initial newborn ABR screening results [13]. Differences in screening methods, timing, anesthetic exposure, and study populations may contribute to these findings. Our study did not include detailed information on anesthetic agents, doses, or incision-to-delivery intervals. Furthermore, the absence of a statistically significant association in a cohort with only seven final refer outcomes should not be interpreted as definitive evidence that delivery mode or anesthesia has no effect.

The post hoc worst-case sensitivity analysis demonstrated the potential influence of incomplete outpatient follow-up on observed associations. Under this hypothetical scenario, lower gestational age remained nominally associated with the expanded refer outcome (*p* = 0.028). General anesthesia compared with regional anesthesia (*p* = 0.014) and Syrian nationality compared with Turkish nationality (*p* = 0.008) also showed nominal associations. These findings do not establish causal relationships or indicate that the incomplete cases had abnormal final screening results. Instead, they demonstrate how assumptions regarding unresolved screening outcomes may alter the results of exploratory comparisons. Because the reasons for incomplete follow-up were not systematically documented, the potential contributions of clinical circumstances, access barriers, and family-related factors could not be determined.

Nationality showed a nominal overall association with final AABR screening outcome (*p* = 0.047). The observed refer proportions were 5.8% (3/52) among Syrian newborns and 1.6% (4/245) among Turkish newborns, whereas no final refer results occurred in the heterogeneous Other nationality group (0/94). Çıkrıkçı et al. also reported differences in AABR screening outcomes between Syrian and Turkish newborns and identified differences in selected perinatal characteristics [14]. However, nationality should not be interpreted as a biological determinant of hearing-screening outcomes. The small number of final refer events, the zero-event category, and the absence of detailed socioeconomic and prenatal care information prevent reliable conclusions regarding the mechanisms underlying these differences. Furthermore, the overall nationality association did not remain statistically significant after Holm correction. The finding should therefore be considered hypothesis-generating.

A central finding of this audit was the distinction between final AABR refer results and protocol-driven audiological referrals despite normal screening. Among the 406 newborns in the total cohort, 48 (11.8%) completed screening and were referred for further audiological assessment: 7 (1.7%) because of a final AABR refer result and 41 (10.1%) because of predefined hearing-loss risk factors despite a normal AABR result. When restricted to the 391 newborns who completed screening, the overall referral proportion was 12.3% (48/391). The remaining 15 newborns (3.7%) had incomplete screening and were not included among the 48 completed-screening referrals because their final screening status remained unresolved. These measures describe different aspects of the screening program and should not be used interchangeably. This distinction is based on the screening results and predefined indications for referral, rather than on observed differences in confirmed hearing-loss outcomes. Because post-referral diagnostic findings were unavailable, the diagnostic yield and positive predictive value of the two referral pathways could not be estimated or compared. In particular, risk-factor-based referrals do not indicate screening failure or hearing impairment. The JCIH 2019 position statement recommends continued audiological evaluation and surveillance for infants with relevant risk factors even when newborn hearing screening is passed [16]. Distinguishing screening outcomes from risk-based follow-up decisions is therefore essential for meaningful interpretation of program performance.

Finally, 15 newborns (3.7% of the total cohort) did not complete outpatient rescreening after an initial refer result. Their final AABR outcomes remained unknown, and the absence of confirmatory diagnostic records from external referral centers further limited assessment of subsequent hearing outcomes. The worst-case sensitivity analysis demonstrated that assumptions about these unresolved cases could change the observed statistical associations. These findings highlight the importance of documenting screening completion, strengthening outpatient recall procedures, and improving communication between birth hospitals and audiological referral services. Such measures may support more reliable program evaluation and timely identification of newborns requiring further assessment [1,16].

### 4.1. Strengths and Limitations

This study has several methodological strengths. It included a consecutive cohort of 406 singleton newborns evaluated under a standardized multi-stage AABR screening protocol. The inclusion of singleton newborns avoided clustering associated with multiple births. Importantly, the cohort was classified into four mutually exclusive outcome groups, distinguishing normal AABR results without referral, normal AABR results with risk-factor-based referral, final AABR refer results, and incomplete outpatient rescreening. This classification prevented risk-based referrals from being misinterpreted as abnormal screening outcomes. Additional strengths include adjustment for multiple comparisons using the Holm–Bonferroni procedure and a post hoc worst-case sensitivity analysis to explore the potential impact of incomplete follow-up.

Several limitations should be acknowledged. First, this was a retrospective, single-center study conducted at a private hospital, which may limit the generalizability of the findings. The retrospective design also restricted the analysis to routinely recorded clinical information, and residual confounding from unmeasured factors cannot be excluded. Although age at the recorded screening assessment was available and is reported in Table 1, detailed information on the timing of individual screening attempts, corrected age at testing in preterm infants, and the complete sequence of repeat assessments was not consistently available. Potential influences of transient middle-ear conditions, including residual fluid or vernix, and environmental factors such as ambient noise could not be systematically assessed. These unmeasured factors may have influenced AABR screening outcomes and contributed to the observed differences across delivery-mode and NICU subgroups. Consequently, the exploratory associations should not be interpreted as causal.

Second, only seven newborns had a final AABR refer result. This small number of events limited statistical power and resulted in imprecise estimates and wide confidence intervals. Sparse-event bias may also have affected odds ratio estimates. The exploratory MDE analysis indicated that, under the specified assumptions, absolute differences of approximately 6.7 percentage points for delivery mode and 13.7 percentage points for anesthesia type would be required to achieve 80% power. These relatively large detectable differences underscore that smaller, potentially clinically relevant associations cannot be excluded. Accordingly, the observed associations should be interpreted as exploratory rather than definitive predictors of screening outcomes.

Third, detailed anesthesia-related information, including specific anesthetic agents, drug dosages, duration of maternal exposure, and incision-to-delivery intervals, was not available. Furthermore, anesthesia type was closely related to delivery mode and may have been confounded by elective versus emergency delivery status, clinical indications, and other obstetric factors that could not be adequately evaluated. Therefore, the study could not assess potential dose-dependent or timing-related effects of maternal anesthesia or estimate an independent association between anesthesia technique and neonatal AABR screening outcomes.

Fourth, 15 newborns did not complete outpatient rescreening, leaving their final screening outcomes unknown. Although a worst-case sensitivity analysis was conducted, this hypothetical approach cannot establish their actual AABR results or fully eliminate potential bias associated with incomplete follow-up.

Finally, confirmatory diagnostic audiological results from specialized referral centers were not available in the hospital database. Consequently, final AABR refer results could not be verified against diagnostic auditory brainstem response testing, and the study could not determine the prevalence or predictors of confirmed permanent hearing loss. The findings therefore relate specifically to screening and referral outcomes rather than diagnostic hearing impairment.

### 4.2. Clinical Implications and Programmatic Recommendations

The findings of this audit highlight two practical priorities for newborn hearing-screening programs. First, screening registries should distinguish infants with final AABR refer results from those who pass screening but require audiological surveillance because of predefined hearing-loss risk factors. These groups have different follow-up needs, and separating them may improve referral coordination, resource allocation, and interpretation of program performance. Importantly, neither a final AABR refer result nor a risk-factor-based referral constitutes a diagnosis of hearing impairment. Follow-up pathways should remain consistent with established newborn hearing-screening recommendations [16]. Given the limited number of final AABR refer outcomes and the exploratory nature of the analyses, the nominal associations observed in this cohort should not be used to modify individual clinical screening or referral decisions.

Second, screening programs should strengthen systems for identifying and following newborns who do not complete outpatient rescreening. In our cohort, 15 infants had unresolved final screening outcomes, demonstrating a potential gap in screening completion. Electronic tracking registries, discharge counseling, appointment reminders, and improved communication with referral centers may help reduce incomplete follow-up. Where language barriers are relevant, multilingual parental information may also support communication and continuity of care. These measures could improve the completeness of screening records and facilitate timely audiological assessment [1,16].

## 5. Conclusions

In this descriptive cohort audit of universal newborn AABR screening, lower gestational age and NICU admission showed nominal exploratory associations with final AABR refer results, whereas only the predefined hearing-loss risk factor composite remained statistically significant after Holm–Bonferroni correction. Delivery mode, maternal anesthesia type, infant sex, and Apgar scores were not significantly associated with the primary screening outcome, although the small number of final refer events limited statistical power.

Importantly, most audiological referrals occurred despite normal AABR results because of predefined hearing-loss risk factors, emphasizing the need to distinguish abnormal screening results from risk-based surveillance. Incomplete outpatient rescreening in 3.7% of newborns further highlighted the importance of systematic follow-up tracking. Larger prospective multicenter studies incorporating confirmatory diagnostic audiological outcomes are needed to clarify the relationships between perinatal characteristics, screening results, and subsequent hearing outcomes.

## Figures and Tables

**Table 1:** Baseline Characteristics of the Study Cohort Stratified by Screening and Referral Outcomes (N = 406).

Characteristic	Group 1: Normal Pass (No Referral) (*n* = 343)	Group 2: Risk Referral (Normal AABR) (*n* = 41)	Group 3: Final AABR Refer (*n* = 7)	Group 4: Incomplete/Lost to Follow-Up (*n* = 15)	Total Cohort (N = 406)
**Maternal Age (years)**					
-Mean ± SD	29.6 ± 5.6	30.6 ± 6.0	29.0 ± 9.2	28.9 ± 6.0	29.6 ± 5.7
-Median [IQR]	29 [25–33]	30 [26–35]	29 [17–36]	29 [23–34]	29 [25–33]
**Gestational Age (weeks)**					
-Mean ± SD	38.5 ± 1.4	37.0 ± 2.1	37.0 ± 1.6	37.8 ± 2.0	38.3 ± 1.6
-Median [IQR]	38 [38–39]	37 [36–38]	37 [36.5–38]	38 [37–39]	38 [38–39]
-<37 weeks, *n* (%)	17 (5.0%)	15 (36.6%)	2 (28.6%)	1 (6.7%)	35 (8.6%)
-37–38 weeks, *n* (%)	165 (48.1%)	21 (51.2%)	4 (57.1%)	9 (60.0%)	199 (49.0%)
-≥39 weeks, *n* (%)	161 (46.9%)	5 (12.2%)	1 (14.3%)	5 (33.3%)	172 (42.4%)
**Birth Weight (g)**					
-Mean ± SD	3307.9 ± 409.7	3009.0 ± 570.7	3012.9 ± 500.2	3201.3 ± 464.8	3268.7 ± 441.0
-Median [IQR]	3330 [3030–3550]	2920 [2650–3440]	2930 [2830–3400]	3410 [2780–3550]	3310 [2965–3540]
**Age at Screening (days)**					
-Mean ± SD	1.4 ± 1.6	10.3 ± 8.1	4.1 ± 4.2	1.9 ± 1.8	2.3 ± 4.1
-Median [IQR]	1 [1–1]	10 [2–14]	1 [1–8]	1 [1–2]	1 [1–1]
**Infant Sex, *n* (%)**					
-Female	178 (51.9%)	16 (39.0%)	2 (28.6%)	9 (60.0%)	205 (50.5%)
-Male	165 (48.1%)	25 (61.0%)	5 (71.4%)	6 (40.0%)	201 (49.5%)
**Delivery Mode, *n* (%)**					
-Vaginal delivery	83 (24.2%)	8 (19.5%)	2 (28.6%)	4 (26.7%)	97 (23.9%)
-Cesarean delivery	260 (75.8%)	33 (80.5%)	5 (71.4%)	11 (73.3%)	309 (76.1%)
**Anesthesia Type, *n* (%)**					
-None (Vaginal)	72 (21.0%)	7 (17.1%)	2 (28.6%)	4 (26.7%)	85 (20.9%)
-Regional (Neuraxial)	248 (72.3%)	30 (73.2%)	5 (71.4%)	6 (40.0%)	289 (71.2%)
-General anesthesia	23 (6.7%)	4 (9.8%)	0 (0.0%)	5 (33.3%)	32 (7.9%)
**1-min Apgar Score**					
-Mean ± SD	9.0 ± 2.2	7.8 ± 2.6	8.9 ± 1.6	7.5 ± 2.8	8.8 ± 2.3
-Median [IQR]	10 [8–10]	8 [7–10]	10 [8–10]	8 [6–10]	10 [8–10]
**5-min Apgar Score**					
-Mean ± SD	9.5 ± 0.6	8.9 ± 1.8	9.6 ± 0.8	8.6 ± 2.5	9.4 ± 1.0
-Median [IQR]	10 [9–10]	10 [8–10]	10 [9.5–10]	9 [9–10]	10 [9–10]
-≤8, *n* (%)	26 (7.6%)	11 (26.8%)	1 (14.3%)	3 (20.0%)	41 (10.1%)
-9, *n* (%)	109 (31.8%)	8 (19.5%)	1 (14.3%)	6 (40.0%)	124 (30.5%)
-10, *n* (%)	208 (60.6%)	22 (53.7%)	5 (71.4%)	6 (40.0%)	241 (59.4%)
**NICU Admission, *n* (%)**					
-No	343 (100.0%)	9 (22.0%)	4 (57.1%)	14 (93.3%)	370 (91.1%)
-Yes	0 (0.0%)	32 (78.0%)	3 (42.9%)	1 (6.7%)	36 (8.9%)
**Predefined Risk Factors, *n* (%)**					
-Absent	343 (100.0%)	0 (0.0%)	3 (42.9%)	14 (93.3%)	360 (88.7%)
-Present (≥1)	0 (0.0%)	41 (100.0%)	4 (57.1%)	1 (6.7%)	46 (11.3%)
**Nationality, *n* (%)**					
-Türkiye	213 (62.1%)	28 (68.3%)	4 (57.1%)	6 (40.0%)	251 (61.8%)
-Syria	47 (13.7%)	2 (4.9%)	3 (42.9%)	5 (33.3%)	57 (14.0%)
-Other nationalities	83 (24.2%)	11 (26.8%)	0 (0.0%)	4 (26.7%)	98 (24.1%)

**Note:** In Group 2 (risk-based referral), all 32 NICU infants were referred: 29 met the JCIH criterion of NICU stay > 5 days, and 3 had shorter stays (2 days) but were referred due to a coexisting family history of hearing loss.

**Table 2:** Association Between Perinatal Characteristics and Final AABR Screening Outcome Among Completed Screens (*n* = 391).

Variable	Normal Pass (*n* = 384)	Final Refer (*n* = 7)	Refer Rate, % (Exact 95% CI)	Unadjusted OR (Exact 95% CI)	*p*-Value
Infant Sex					
Female	194	2	1.02 [0.12–3.64]	Reference	—
Male	190	5	2.56 [0.84–5.88]	2.55 (0.41–27.05)	0.284
Delivery Mode					
Vaginal	91	2	2.15 [0.26–7.55]	Reference	—
Cesarean	293	5	1.68 [0.55–3.87]	0.78 (0.12–8.29)	0.673
Anesthesia Type					
Regional	278	5	1.77 [0.58–4.07]	Reference	—
General	27	0	0.00 [0.00–12.77]	Not estimable	1.000
None (vaginal)	79	2	2.47 [0.30–8.64]	1.41 (0.13–8.79)	0.648
Overall anesthesia comparison	—	—	—	—	0.791 *
NICU Admission					
No	352	4	1.12 [0.31–2.85]	Reference	—
Yes	32	3	8.57 [1.80–23.06]	8.16 (1.15–50.55)	**0.018**
Predefined Risk Factors					
No risk factors	343	3	0.87 [0.18–2.51]	Reference	—
≥1 risk factor	41	4	8.89 [2.48–21.22]	11.02 (1.80–77.88)	**0.004**
Nationality					
Türkiye	241	4	1.63 [0.45–4.13]	Reference	—
Syria	49	3	5.77 [1.21–15.95]	3.67 (0.52–22.42)	0.106
Other	94	0	0.00 [0.00–3.85]	Not estimable	—
Overall nationality comparison	—	—	—	—	0.047 *
Gestational Age Categories					
<37 weeks	32	2	5.88 [0.72–19.68]	10.38 (0.64–112.5)	—
37–38 weeks	186	4	2.11 [0.58–5.30]	3.57 (0.37–34.5)	—
≥39 weeks	166	1	0.60 [0.02–3.29]	Reference	—
Overall gestational age category comparison	—	—	—	—	0.052 *
Continuous Variables, Median [IQR]					
Maternal age, years	29 [26–33]	29 [17–36]	—	—	0.927
Gestational age, weeks	38 [38–39]	37 [36.5–38]	—	—	**0.026**
Birth weight, g	3310 [2980–3540]	2930 [2830–3400]	—	—	0.138
1-min Apgar score	10 [8–10]	10 [8–10]	—	—	0.887
5-min Apgar score	10 [9–10]	10 [9.5–10]	—	—	0.706

**Notes:** AABR, automated auditory brainstem response; NICU, neonatal intensive care unit; OR, odds ratio; CI, confidence interval; IQR, interquartile range. Refer rates were calculated within each category, and exact two-sided 95% confidence intervals for proportions were calculated using the Clopper–Pearson method. Odds ratios and exact 95% confidence intervals were estimated using conditional maximum likelihood where estimable. Categorical variables were compared using Fisher’s exact test for 2 × 2 tables or the Fisher–Freeman–Halton exact test for larger contingency tables, as appropriate. Continuous and ordinal variables were compared using the two-sided Mann–Whitney U test, with asymptotic *p*-values adjusted for tied ranks. An asterisk (*) indicates an overall exact test across all categories. Category-specific *p*-values for anesthesia type and nationality are shown separately from their overall tests. Bold *p*-values indicate unadjusted statistical significance (*p* < 0.05); these findings should be interpreted as exploratory unless they remain significant after multiplicity adjustment. The Holm–Bonferroni procedure was applied to 12 primary comparisons: infant sex, delivery mode, overall anesthesia type, NICU admission, predefined risk factors, overall nationality, gestational age categories, maternal age, continuous gestational age, birth weight, 1-min Apgar score, and 5-min Apgar score. Only the predefined risk factor composite remained significant after multiplicity adjustment (adjusted *p* ≈ 0.048).

**Table 3:** Distribution of Referral Criteria Among Newborns with Normal AABR Results Referred for Risk Factors (*n* = 41).

Predefined Referral Criterion	Number of Infants (*n*)	Proportion (%)
NICU-related prolonged hospitalization (>5 days)	29	70.7
Family history of permanent childhood hearing loss	7	17.1
Craniofacial anomaly (preauricular pit/tag)	3	7.3
Ototoxic medication exposure (>5 days)	1	2.4
Mechanical ventilation (>5 days)	1	2.4
Maternal intrauterine infection/maternal disease	1	2.4
Syndromic disorder	1	2.4

**Note:** NICU, neonatal intensive care unit; AABR, automated auditory brainstem response. Percentages were calculated using the 41 newborns referred because of predefined risk factors as the denominator. Referral criteria were not mutually exclusive, as some newborns had multiple coexisting risk factors; therefore, percentages do not sum to 100%. All newborns in this subgroup had normal bilateral AABR screening results.

**Table 4:** Post Hoc Worst-Case Sensitivity Analysis Assuming All 15 Incomplete Screening Cases Were Final AABR Referrals.

Comparison	Nominal *p*-Value
Gestational age, weeks (continuous)	**0.028**
NICU admission (yes vs. no)	0.120
≥1 predefined risk factor (yes vs. no)	0.090
General vs. regional anesthesia	**0.014**
Syrian vs. Turkish nationality	**0.008**

**Note:** AABR, automated auditory brainstem response; NICU, neonatal intensive care unit. The primary analysis included 384 newborns with a normal AABR result and 7 with a final AABR refer result. For this exploratory worst-case sensitivity analysis, all 15 newborns with incomplete outpatient rescreening were hypothetically classified as final AABR refer cases (22 assumed refers vs. 384 normal passes; N = 406). NICU admission and predefined risk factors were assessed using two-sided Fisher exact tests; the anesthesia comparison was restricted to general versus regional anesthesia, and the nationality comparison to Syrian versus Turkish newborns. Gestational age was compared using the two-sided Mann–Whitney U test with an asymptotic *p*-value adjusted for tied ranks. All *p*-values are nominal and unadjusted for multiple testing. Bold *p*-values indicate nominal statistical significance (*p* < 0.05). Hypothetical referral does not indicate observed screening failure or confirmed hearing impairment.

## Data Availability

The individual-level clinical data underlying this study are not publicly available because they contain sensitive patient information and have not been prepared for public release through an appropriate de-identification process. Requests for access to the data may be directed to the corresponding author and will be considered subject to applicable ethical, institutional, and data-protection requirements.
